# Estimation of genetic variation in yield, its contributing characters and capsaicin content of *Capsicum chinense* Jacq. (ghost pepper) germplasm from Northeast India

**DOI:** 10.7717/peerj.15521

**Published:** 2023-06-22

**Authors:** Joyashree Baruah, Sunita Munda, Neelav Sarma, Twahira Begum, Sudin Kumar Pandey, Sanjoy Kumar Chanda, G. Narahari Sastry, Mohan Lal

**Affiliations:** 1 Department of Botany, Eastern Karbi Anglong College, Sarihajan, Assam, India; 2 Agrotechnology and Rural Development Division, CSIR-North East Institute of Science and Technology, Jorhat, Assam, India; 3 Advances Computation and Data Science Division, CSIR-North East Institute of Science and Technology, Jorhat, Assam, India

**Keywords:** *Capsicum chinense*, Genotypic coefficient of variation, Phenotypic coefficient of variation, Levene’s homogeneity test of variance, Capsaicin

## Abstract

*Capsicum chinense* Jacq. (ghost pepper), a naturally occurring chili species of Northeast India is known throughout the world for its high pungency and a pleasant aroma. The economic importance is due to the high capsaicinoid levels, the main source for pharmaceutical industries. The present study focused on identifying important traits necessary for increasing the yield and pungency of ghost pepper and to determine the parameters for the selection of superior genotypes. A total of 120 genotypes with more than 1.2% capsaicin content (>1,92,000 Scoville Heat Unit, w/w on dry weight basis) collected from different northeast Indian regions were subjected to variability, divergence and correlation studies. Levene’s homogeneity test of variance studied for three environments did not show significant deviation and so homogeneity of variance was reasonably met for analysis of variance study. Genotypic and phenotypic coefficient of variation was highest for fruit yield per plant (33.702, 36.200, respectively), followed by number of fruits per plant (29.583, 33.014, respectively) and capsaicin content (25.283, 26.362, respectively). The trait number of fruits per plant had maximum direct contribution to fruit yield per plant and the trait fruit yield per plant towards capsaicin content in the correlation study. High heritability with high genetic advance, which is the most favored selection criteria was observed for fruit yield per plant, number of fruits per plant, capsaicin content, fruit length and fruit girth. The genetic divergence study partitioned the genotypes into 20 clusters, where fruit yield per plant contributed maximum towards total divergence. Principal components analysis (PCA) studied to determine the largest contributor of variation showed 73.48% of the total variability, of which the PC1 and PC2 contributed 34.59% and 16.81% respectively.

## Introduction

The northeast region of India is a home to rich diversity of *Capsicum* domesticated species and among them *C. chinense* Jacq. (ghost pepper or bhut jolokia or Naga King chili) is immensely popular for its unique aroma and high pungency (Sarwa et al., 2013; Baruah et al., 2019). The region with unique ecological conditions and high humidity, acting as centre of speciation has given rise to the hottest pepper in the world (Guinness Book of World Records, 2007). The genus *Capsicum* (family Solanaceae), a new world crop is represented by thirty-five species (Barchenger, Naresh & Kumar, 2019). It was introduced to India at the end of the 17th century by Portuguese explorers and to Northeast India by Christian missionaries (Basu & De, 2003). Ghost pepper, a semi-perennial species, is a naturally occurring variety of Northeast India (Verma et al., 2013; Baruah & Lal, 2020). Besides its use as a flavoring agent, the capsaicin extracted from this plant species has many pharmacological applications (Meghvansi et al., 2010).

Ghost pepper shows wide variation in morphological characteristics (Purkayastha et al., 2012). According to literature reports, ghost pepper has higher capsaicin concentration than other Indian chili varieties (Baruah et al., 2014; Baruah et al., 2019), making it a potential crop for the extraction of oleoresin capsicum and capsaicin for commercial uses (Meghvansi et al., 2010; Baruah & Lal, 2020; Bhandari et al., 2021). Low capsaicin-yielding varieties are not suitable for commercial cultivation because of the bottleneck associated with a high cost of capsaicin extraction (Baruah & Lal, 2020). However, despite its advantages, no measures were taken for the improvement of this important crop as the production is not up to the demand to meet the quality requirements, such as high fruit yield, high capsaicin, *etc*. It may be due to the lack of superior varieties available in the public domain or the use of low-quality capsaicin content lines for cultivation. To overcome this, information on morphological characteristics, evaluation of a large number of germplasm, diversity study and creation of gene bank is pre-requisite, allowing the exploration of genetic variability efficiently (Santos-Pessoa et al., 2018; Karim et al., 2022). Heritability criteria determine the extent to which it is transmissible from parents to offspring and is mostly preferred when used in association with other parameters (Afiah, Mohammad & Saleem, 2000; Munda, Dutta & Lal, 2021; Begum et al., 2022). Path coefficient analysis helps in identifying useful traits associated with yield based on the direct and indirect effect of the traits on economical traits (Seyoum, Alamerew & Bantte, 2012; Pandey et al., 2022). Also, to select suitable advanced cultivars within a short period of time, the information on all the genetic parameters will be very much helpful for the breeder (Dutta et al., 2017; Karim et al., 2022). The variability present in the germplasm is used for effective selection of diverge parents, which in turn would be helpful to obtain hybrids with greater heterotic effect (Correa & Gonçalves, 2012). The degree of genetic variability can be measured using Mahalanobi’s D^2^ analysis, which is a powerful tool for determining the relative contribution of different traits (both inter and intra cluster level) on total divergence in self-pollinated plants (Hasan et al., 2015; Munda, Dutta & Lal, 2021). The knowledge of morphological as well as genetic diversity is very much essential for initiating any breeding programme which focuses on the development of superior varieties. Many reports were available in the public domain regarding the variability and diversity study of different *Capsicum* species. However, very few reports were accessible regarding this important plant with small population, which cannot be considered reliable as small sample size often produces skewed results (Baruah et al., 2019). So, there is a need for proper scientific study on genetic variability, heritability, genetic advance, and interrelationship among economically important traits along with their direct and indirect effect on fruit yield and capsaicin content through path studies. Therefore, a planned breeding experiment was conducted to identify the selection criteria to develop high-yielding and higher capsaicin content lines of this industrially important crop.

## Materials & Methods

### Planting material and experimental design

The experiment was carried out at CSIR-NEIST (North East Institute of Science and Technology) experimental farm, Jorhat, Assam, India (26°44′N, 94°9′E, 94 m a.s.l.). One hundred and twenty (120) genotypes with more than 1.2% (1,92,000 SHU) capsaicin content on dry weight basis were selected from an initial collection of 227 germplasm (Baruah et al., 2019), which were planted in randomized complete block design (RCBD) with three replications during three consecutive years *i.e., kharif* 2017, *kharif* 2018 and *kharif* 2019. Among them genotypes-RRL-BJ-102 and 18 were brown variants and RRL-BJ-20 and 25 were yellow variants while RRL-BJ-92 and 58 were round-shaped red variants (Fig. S1). For capsaicin estimation, fully ripe chilies per plot were harvested, followed by immediate drying to retain their quality, such as intact red colour, pungency, *etc*. A total of 16 plants from each genotype were planted in a plot size of 2.5 × 3 m, with 60 × 60 cm plant-plant and line-line spacing. As recommended fertilizer dose (NPK) of 120:80:60 kg/ha/year was applied in the experiment. All standard agronomical practices were followed to raise a good crop. Morpho-agronomic characterization was done based on IPGRI (International Plant Genetic Resources Institute, 1995) report on *Capsicum* species. For all the studied traits, morphological data were collected in triplicate during *kharif* 2017 and their average was calculated. The same process was followed during *kharif* 2018 and 2019. The data obtained from three years were then pooled, and their average value was taken for final statistical analysis. Meteorological data recorded during the study years was presented in Table S1.

**Figure 1:**
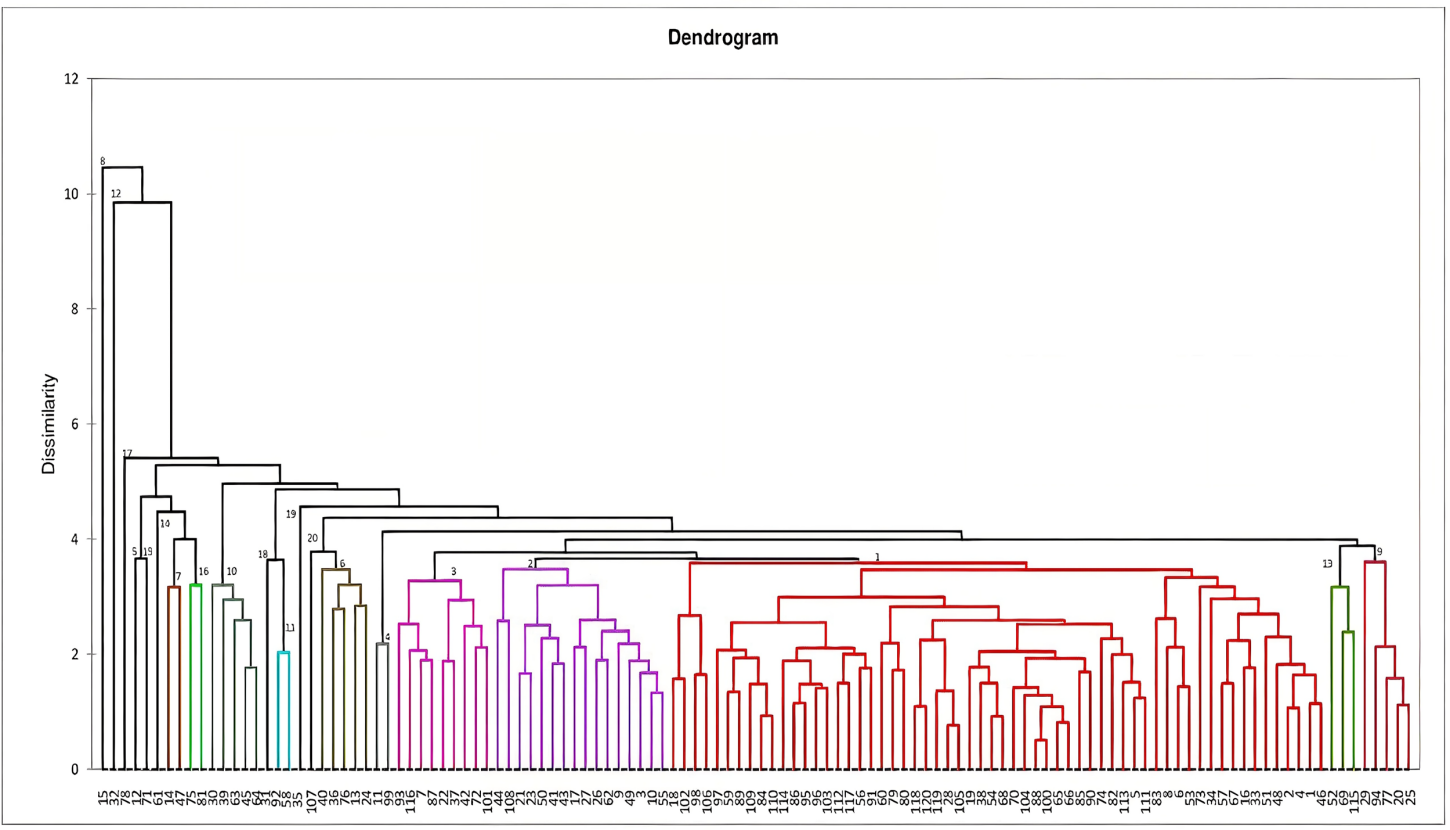
Dendrogram constructed based on Mahalanobi’s distance matrix and unweighted pair-group method to determine the genotypes clustering for morphological characters.

### Traits studied

Data were recorded for 11 traits, *viz*-vegetative plant height (cm), number of main branch, leaf length (cm), leaf breadth (cm), fruit length (cm), fruit girth (cm), number of fruits per plant, fruit yield per plant (g), capsaicin content percent, days to 50% flowering and days to maturity for three consecutive years (Table S2). After harvesting the mature fruits were oven dried (45 °C for 3–4 days depending on fruit thickness) for extraction of capsaicin content. The estimation of capsaicin was done using a spectrophotometric method in triplicates (Thimmaiah, 1999), followed by their validation using uHPLC method. Two grams of dried chilli powder was dissolved in 4 mL of ethanol extract and kept in a water bath at 80 °C for 3 h, manually inverted after every hour. The samples were then kept in room temperature for cooling. The supernatant layer of each sample was filtered through Nylon 33 mm 0.45 µm filter (AxivaSchem. Pvt. Ltd., Sonipat, India). A uHPLC Ultimate 3000 (Thermo Fisher Scientific, Waltham, MA, USA) system equipped with Betasil C_18_ column (particle size 3 µm, dimension 150 × 4.6 mm) was used for analysis, maintaining column temperature at 60 °C, sampler temperature at 20 °C and sample volume: 5 µL. A binary mixture of water-acetonitrile at a 50:50 ratio was used as mobile phase and the flow rate was 1.5 mL/min. The procedure for capsaicin estimation described by Bhandari et al. (2021) was used in the study.

### Statistical analysis

To confirm the homogeneity of the studied environments Levene’s test (1960) was performed using SPSS software (version 16.03) before pooling the data, which is given as-

H_O_: ${\sigma }_{1}^{2}={\sigma }_{2}^{2}=\ldots ={\sigma }_{k}^{2}$

H_a_: ${\sigma }_{i}^{2}\not = {\sigma }_{j}^{2}$ for at least one pair.

For ‘Y’ variable with ‘N’ sample size having ‘k’ subgroups, Levene’s test is statistically defined as- $W= \frac{(N-k)}{(k-1)} \frac{{\mathop{\sum }\nolimits }_{i=1}^{k}{N}_{i}({\overline{Z}}_{i.}-{\overline{Z}}_{..})^{2}}{{\mathop{\sum }\nolimits }_{i=1}^{k}{\mathop{\sum }\nolimits }_{j=1}^{{N}_{i}}({Z}_{ij}-{\overline{Z}}_{i.})^{2}} $

where ‘*Ni*’ is the sample size of ith subgroup.

*Z*_*ij*_ = |*Yij* − *Yi*.|, where *Yi*. is either the mean or median of the *i*th subgroup.

Statistical analyses were performed using INDOSTAT software version 8.2. The data were subjected to a standard statistical method of Analysis of variance (ANOVA) for RCBD (Panse & Sukhatme, 1978). Genotypic and phenotypic coefficients of variability (GCV, PCV) were calculated following the method proposed by Burton & De-Vane (1953); Broad sense heritability (H_bs_) was computed following method suggested by Allard (1960); correlation coefficient by Fisher (1954); genetic advance as per the method given by Johnson, Robinson & Comstock (1955) and path coefficient analysis by Dewey & Lu (1959). XLSTAT software was used for clustering analysis and principal component analysis based on Mahalanobi (1936) distance matrix and unweighted pair-group method. The genetic parameters were calculated using the following equation as follows-

1. Genotypic coefficients of variation (GCV) = $ \frac{\sqrt{GV}}{\overline{X}} \times 100$
2. Phenotypic coefficients of variation (PCV) = $ \frac{\sqrt{PV}}{\overline{X}} \times 100$,
3. Heritability in broad sense (H_bs_) = $ \frac{GV}{PV} \times 100$, where, GV = Genotypic variance, PV = Phenotypic variance, X = mean of the character
4. Genetic advance as percent mean (GA) = $K\times \frac{GV}{PV} \times 100$, where, K = standardized selection differential ( *K* = 2.06 at 5% selection intensity).

## Results

Levene’s test of homogeneity of variance (1960) studied for three environments presented in Table 1, where the traits did not show any significant deviation ( *P* ≤ 0.005) over the environments and hence homogeneity of variance assumption is reasonably met for one-way ANOVA. Further ANOVA analysis (Table 2) performed for all the traits showed highly significant differences in the studied genotypes at *P* ≤ 0.005.

**Table 1:** Levene’s test of homogeneity computed for three environments.

Characters	Levene Statistic	DF1	DF2	*α* value
Plant height (cm)	0.046	2	357	0.955
No of main branch	1.373	2	357	0.255
Leaf length (cm)	0.272	2	357	0.762
Leaf breadth (cm)	0.548	2	357	0.579
Days to 50% flowering	1.283	2	357	0.278
Capsaicin content (%)	0.145	2	357	0.865
Fruit length (cm)	0.259	2	357	0.772
Fruit girth (cm)	0.945	2	357	0.390
Fruit yield/plant (g)	0.017	2	357	0.984
No of fruits/plant	0.423	2	357	0.656
Days to maturity	1.852	2	357	0.158

**Notes.**

DF Degree of freedom *α* Significance at *P* ≤ 0.005 (0.5%)

**Table 2:** Analysis of variance (ANOVA) and estimation of genetic parameters for 120 genotypes of *C. chinense* Jacq.

Characters	ANOVA			Genetic parameters				Mean performance				
	Replication (DF = 2)	Treatment (DF = 119)	Error (DF = 238)	GCV	PCV	H^2^ (Broad Sense)	GA 5%	General Mean	CV%	SE	CD 5%	Range
Plant height (cm)	1812.817	103.967^*^	23.119	8.609	11.735	53.824	13.011	60.299	7.974	3.926	7.739	42.777–75.477
No of main branch	7.284	1.214^*^	0.463	12.138	20.486	35.103	14.814	4.123	16.503	0.556	1.095	2.777–5.777
Leaf length (cm)	81.737	3.315^*^	0.527	11.117	13.917	63.810	18.294	8.672	8.372	0.593	1.168	5.547–10.603
Leaf breadth (cm)	65.284	2.011^*^	0.407	14.762	19.596	56.750	22.909	4.953	23.652	0.521	1.905	2.710–6.580
Days to 50% flowering	1533.333	86.141^*^	4.864	7.053	7.660	84.780	13.378	73.799	2.988	1.801	3.550	65.110–91.110
Capsaicin content (%)	2.100	0.874^*^	0.025	25.283	26.362	91.987	49.953	2.105	7.532	0.128	0.255	1.327–4.187
Fruit length (cm)	21.823	2.531^*^	0.066	13.869	14.417	92.543	27.485	6.536	3.937	0.210	0.414	4.590–8.83
Fruit girth (cm)	21.619	2.658^*^	0.076	13.734	14.331	91.840	27.113	6.755	15.259	0.226	1.673	4.087–8.733
Fruit yield/plant (g)	116631.064	62121.563^*^	3027.908	33.702	36.200	86.676	64.636	416.437	13.213	44.929	88.566	188.627–877.060
No of fruits/plant	2246.073	1116.781^*^	84.437	29.583	33.014	80.297	54.609	62.706	10.655	5.425	10.694	32.777–110.577
Days to maturity	1598.982	63.686^*^	5.244	2.588	2.915	78.789	4.732	170.566	1.339	1.870	3.675	157–180.333

**Notes.**

* *P* ≤ 0.005 (0.5%).

DF Degree of freedom GCV Genotypic coefficient of variation PCV Phenotypic coefficient of variation GA Genetic advance CV Critical variance SE Standard error CD Critical difference

Genetic variability parameters evaluated for all the germplasm are presented in Table 2. The highest genotypic and phenotypic variation coefficients (GCV, PCV) were recorded for fruit yield per plant (33.702, 36.200). It was followed by number of fruits per plant (29.583, 33.014), capsaicin content (25.283, 26.362), leaf breadth (14.762, 19.596), fruit length (13.869, 14.417) and fruit girth (13.734, 14.331). The lowest GCV and PCV estimates were recorded for traits-days to maturity (2.588, 2.915), days to 50% flowering (7.053, 7.660) and plant height (8.609, 11.735). All the studied traits showed lower GCV estimates than PCV, except for days to 50% flowering and days to maturity, where least difference in magnitude was observed. The highest difference between GCV-PCV estimates was observed for traits-plant height, number of main branch, leaf length and breadth, fruit length and fruit girth, fruit yield per plant and number of fruits per plant. High heritability (H_bs_) was observed for fruit length (92.543), capsaicin content (91.987), fruit girth (91.840), fruit yield per plant (86.676), days to 50% flowering (84.780) and number of fruits per plant (80.297). Moderate heritability was observed for traits days to maturity (78.789), leaf length (63.810), leaf breadth (56.750) and plant height (53.824) while low heritability was depicted in number of main branch (35.103). High genetic advance (%) was observed for fruit yield per plant (64.636), followed by number of fruits per plant (54.609), capsaicin content (49.953), fruit length (27.485) and girth (27.113) and leaf breadth (22.909). While, high heritability with high genetic advance, was observed for the traits-fruit yield per plant (64.636), number of fruits per plant (54.609), capsaicin content (49.953), fruit length (27.485) and fruit girth (27.113) and moderate heritability with high genetic advance for leaf breadth (22.909).

The genotypic and phenotypic correlation coefficient for different traits is presented in Table 3. For the studied traits, the genotypic correlation was found higher than the phenotypic correlation, indicating strong inherent relation. At both genotypic and phenotypic level (significant at *P* ≤ 0.01), fruit yield per plant showed a positive and highly significant correlation with number of fruits per plant (0.880, 0.825, respectively), plant height (0.639, 0.569, respectively), capsaicin content (0.475, 0.437, respectively), days to 50% flowering (0.460, 0.412, respectively), fruit length (0.454, 0.451, respectively), fruit girth (0.417, 0.408, respectively), number of main branch (0.383, 0.185, respectively), leaf breadth (0.358, 0.250, respectively) and leaf length (0.311, 0.225, respectively). Capsaicin content showed positive and significant correlation (significant at *P* ≤ 0.01) with days to 50% flowering (0.622, 0.555, respectively), fruit yield per plant (0.475, 0.437, respectively), days to maturity (0.529, 0.517, respectively), number of fruits per plant (0.356, 0.315, respectively), plant height (0.237, 0.239, respectively), fruit girth (0.292, 0.276, respectively) and length (0.253, 0.242, respectively). Number of fruits per plant also showed positive and highly significant genotypic and phenotypic correlation with plant height (0.368, 0.217, respectively), number of main branch (0.284, 0.155, respectively) and days to 50% flowering (0.312, 0.277, respectively).

**Table 3:** Genotypic and phenotypic correlation matrix for 11 characteristics of 120 *C. chinense* Jacq. genotypes from pooled data.

Characters	Plant height (cm)	No of main branch	Leaf length (cm)	Leaf breadth (cm)	Days to 50% flowering	Capsaicin content (%)	Fruit length (cm)	Fruit girth (cm)	Fruit yield/ plant (g)	No of fruits/ plant	Days to maturity
Plant height (cm) G	1.000										
P	1.000										
No of main branch G	0.258^**^	1.000									
P	−0.124^*^	1.000									
Leaf length (cm) G	0.435^**^	0.239^**^	1.000								
P	0.306^**^	0.090	1.000								
Leaf breadth (cm) G	0.429^**^	0.156^**^	0.963^**^	1.000							
P	0.299^**^	0.049	0.933^**^	1.000							
Days to 50% flowering G	0.301^**^	0.257^**^	0.242^**^	0.228^**^	1.000						
P	0.230^**^	0.141^**^	0.204^**^	0.187^**^	1.000						
Capsaicin content (%) G	0.237^**^	0.162^**^	0.008	0.076	0.622^**^	1.000					
P	0.239^**^	0.085	0.058	0.052	0.555^**^	1.000					
Fruit length (cm) G	0.535^**^	0.216^**^	0.357^**^	0.337^**^	0.288^**^	0.253^**^	1.000				
P	0.360^**^	0.130^*^	0.278^**^	0.254^**^	0271^**^	0.242^**^	1.000				
Fruit girth (cm) G	0.485^**^	0.271^**^	0.540^**^	0.522^**^	0.324^**^	0.292^**^	0.826^**^	1.000			
P	0.324^**^	0.163^**^	0.415^**^	0.385^**^	0.301^**^	0.276^**^	0.828^**^	1.000			
Fruit yield/plant (g) G	0.639^**^	0.383^**^	0.311^**^	0.358^**^	0.460^**^	0.475^**^	0.454^**^	0.417^**^	1.000		
P	0.569^**^	0.185^**^	0.225^**^	0.250^**^	0.412^**^	0.437^**^	0.451^**^	0.408^**^	1.000		
No of fruits/plant G	0.368^**^	0.284^**^	0.092	0.162^**^	0.312^**^	0.356^**^	0.009	−0.030	0.880^**^	1.000	
P	0.217^**^	0.155^**^	0.043	0.094	0.277^**^	0.315^**^	0.025	−0.008	0.825^**^	1.000	
Days to maturity G	0.199^**^	0.213^**^	−0.017	−0.041	0.555^**^	0.529^**^	0.114^**^	0.146^**^	0.160^**^	0.067	1.000
P	0.183^**^	0.094	0.023	0.014	0.571^**^	0.517^**^	0.113^*^	0.136^**^	0.152^**^	0.079	1.000

**Notes.**

* *P* ≤ 0.05 (5%).

** *P* ≤ 0.01 (1%) respectively.

G genotypic correlation P phenotypic correlation

Genotypic path study for fruit yield per plant was analyzed and presented in Table 4 where number of fruits per plant (0.880) showed maximum positive direct effect on fruit yield per plant, followed by fruit length (0.274) and fruit girth (0.200). Direct positive effects of other traits were very negligible with low residual effects (0.298), which indicates that the traits taken for the study have 70% accuracy for yield determination in ghost pepper. Path analysis for capsaicin content (Table 5) showed that highest positive direct effect on the trait was shown by fruit yield per plant (2.375), followed by days to 50% flowering (0.273), days to maturity (0.202) and plant height (0.115) (Table 4). Leaf length showed very weak direct association (0.033) and rest of the traits showed direct negative effect. The number of fruits per plant showed maximum negative direct effect (−1.802) towards capsaicin content, indicating that both these characters cannot be improved simultaneously. Indirect contribution to capsaicin content was shown by days to 50% flowering *via* fruit yield per plant (1.092) and days to maturity (0.118). Fruit length also showed indirect contribution towards capsaicin content *via* plant height (0.108). Similarly, fruit yield per plant showed indirect contribution *via* plant height (0.114) and days to 50% flowering (0.125). Plant height also contributed to capsaicin content indirectly *via* fruit yield per plant (1.338). A residual effect of 0.504 was obtained which indicated that the studied traits have 50% accuracy for capsaicin estimation in ghost pepper.

**Table 4:** Genotypic path coefficient table showing direct and indirect effects of different traits on fruit yield/plant (g).

Characters	Plant height (cm)	No of main branch	Leaf length (cm)	Leaf breadth (cm)	Days to 50% flowering	Capsaicin content (%)	Fruit length (cm)	Fruit girth (cm)	No. of fruits/ plant	Days to maturity	Fruit yield/ plant (g)
Plant height (cm)	**0.039**	0.001	0.028	−0.013	0.004	0.008	0.146	0.097	0.324	0.006	0.639^*^
No of main branch	−0.010	**0.005**	0.015	−0.005	0.003	0.004	0.059	0.054	0.250	0.007	0.383^*^
Leaf length (cm)	−0.017	0.001	**0.064**	−0.029	0.003	0.002	0.098	0.108	0.081	−0.001	0.311^*^
Leaf breadth (cm)	−0.017	0.001	0.062	**−0.030**	0.003	0.002	0.092	0.104	0.142	−0.001	0.358^*^
Days to 50% flowering	−0.012	0.001	0.016	−0.007	**0.013**	0.013	0.079	0.065	0.274	0.017	0.460^*^
Capsaicin content (%)	−0.013	0.001	0.005	−0.002	0.007	**0.025**	0.069	0.058	0.314	0.011	0.475^*^
Fruit length (cm)	−0.021	0.010	0.023	−0.010	0.004	0.006	**0.274**	0.165	0.008	0.004	0.454^*^
Fruit girth (cm)	−0.019	0.001	0.035	−0.016	0.004	0.007	0.226	**0.200**	−0.026	0.005	0.417^*^
No of fruits/plant	−0.014	0.001	0.006	−0.005	0.004	0.009	0.003	−0.006	**0.880**	0.002	0.880^*^
Days to maturity	−0.008	0.001	−0.001	0.001	0.007	0.009	0.031	0.029	0.059	**0.031**	0.160^*^

**Notes.**

Residual effects: 0.298.

* Indicates significance at 1% level.

Bold values indicate direct effects.

**Table 5:** Genotypic path coefficient table showing direct and indirect effect of different traits on capsaicin content (%).

Characters	Plant height (cm)	No of main branch	Leaf length (cm)	Leaf breadth (cm)	Days to 50% flowering	Fruit length (cm)	Fruit girth (cm)	Fruit yield/ plant (g)	No of fruits/ plant	Days to maturity	Capsaicin content (%)
Plant height(cm)	**0.115**	−0.033	0.050	−0.170	0.082	−0.415	−0.059	1.338	−0.664	0.007	0.237^*^
No of main branch	0.052	**−0.129**	0.027	−0.062	0.070	−0.168	−0.033	0.909	−0.511	0.007	0.162^*^
Leaf length(cm)	0.088	−0.031	**0.033**	−0.381	0.066	−0.277	−0.066	0.738	−0.166	−0.001	0.008
Leaf breadth(cm)	0.110	−0.020	0.087	**−0.396**	0.062	−0.262	−0.064	0.851	−0.292	−0.001	0.076
Days to 50% flowering	0.061	−0.033	0.028	−0.090	**0.273**	−0.224	−0.040	1.092	−0.562	0.118	0.622^*^
Fruit length(cm)	0.108	−0.028	0.041	−0.133	0.079	**−0.776**	−0.101	1.077	−0.017	0.004	0.253^*^
Fruit girth (cm)	0.098	−0.035	0.062	−0.207	0.088	−0.641	**−0.123**	0.990	0.054	0.005	0.292^*^
Fruit yield/plant (g)	0.114	−0.049	0.036	−0.142	0.125	−0.352	−0.051	**2.375**	−1.586	0.005	0.475^*^
No of fruits/plant	0.074	−0.037	0.010	−0.064	0.085	−0.007	0.004	2.090	**−1.802**	0.002	0.356^*^
Days to maturity	0.040	−0.027	−0.002	0.016	0.151	−0.089	−0.018	0.381	−0.120	**0.202**	0.529^*^

**Notes.**

Residual value: 0.50402.

* *P* ≤ 0.01 (1%) level.

Bold values indicate direct effects.

Dendrogram constructed for genetic divergence study using Mahalanobi’s D^2^ analysis are shown in Fig. 1. Based on the degree of divergence the genotypes were grouped into 20 clusters. Cluster I consists of highest number of genotypes (60), followed by cluster II (16) and 3 (9), while nine genotypes came out as distinct entity. For most of the genotypes, the grouping is in accordance with their morphological characteristics, *viz*-RRL-BJ-102 and 18 (brown variants), RRL-BJ-20 and 25 (yellow variants), RRL-BJ-92 and 58 (round red variants) (Fig. S1). According to Mahalanobi’s distance matrix average intra-cluster divergence ranged from 0 to 112.11 (Table 6) with maximum distance observed within cluster I (60 genotypes) and minimum for cluster V, XII, XIV, VIII, XV, XVIII, XVII, XIX and XX (0.00). This indicates the presence of wide genetic diversity among the genotypes of the cluster, while the genotypes in solitary clusters can serve as potent parents owing to their diverge traits, which separates them from genotypes in other clusters. Inter-cluster divergence ranged from 11.48–691.79, with highest distance (691.79) seen between cluster I and XIX, followed by cluster XV and XIX (689.64), cluster VII and XIX (677.99) and cluster XVI and XIX (663.10). Minimum intercluster distance (11.48) was observed between cluster III and XIII.

**Table 6:** Average intra and inter-cluster distances values (Mahalanobi’s D^2^) for 20 clusters in *C. chinense* Jacq. genotypes using pooled data.

	I	II	III	IV	V	VI	VII	VIII	IX	X	XI	XII	XIII	XIV	XV	XVI	XVII	XVIII	XIX	XX
I	112.11	137.733	150.048	112.892	262.486	222.660	288.027	128.520	84.072	38.201	247.272	159.776	150.988	301.766	299.643	273.028	217.098	206.807	691.791	14.274
II		64.580	14.868	26.561	124.843	86.389	151.094	15.437	59.832	117.918	110.157	24.499	14.779	164.776	162.378	136.287	80.777	71.136	527.584	127.390
III			68.606	39.148	112.799	73.709	138.587	24.959	72.000	130.215	98.100	16.552	11.482	151.984	150.204	123.744	69.443	59.471	539.959	139.390
IV				108.224	150.030	110.727	175.537	20.153	37.674	93.891	134.505	47.688	39.201	189.620	186.970	160.678	104.566	94.310	502.696	103.176
V					0.00	42.973	29.692	134.692	181.773	240.323	19.704	103.844	111.962	42.100	38.761	19.314	48.375	58.815	652.408	252.017
VI						12.747	65.889	94.620	141.453	199.736	29.926	66.584	74.417	79.466	78.169	50.838	16.849	21.915	612.724	212.403
VII							12.805	160.040	207.049	265.322	42.298	129.685	138.424	17.188	15.963	17.295	72.600	82.454	677.993	277.758
VIII								0.00	48.745	107.044	119.928	36.457	27.572	173.729	171.745	145.044	89.646	79.832	518.525	118.484
IX									42.504	58.801	167.243	82.820	72.773	220.683	218.398	191.599	135.772	125.932	472.868	75.593
X										50.967	225.696	140.731	131.061	278.974	276.723	249.815	194.113	184.137	416.258	38.263
XI											2.130	88.215	97.228	57.388	53.453	30.188	33.511	42.797	637.014	237.055
XII												0.00	14.389	143.826	141.035	115.399	60.486	50.850	549.211	149.548
XIII													42.646	152.134	149.275	123.479	67.732	58.216	540.700	140.629
XIV														0.00	15.198	30.450	87.152	96.971	390.137	291.217
XV															0.00	28.217	83.112	93.454	689.641	289.384
XVI																13.924	57.353	67.146	663.101	262.774
XVII																	0.00	12.338	606.995	207.216
XVIII																		0.00	596.651	196.945
XIX																			0.00	400.766
XX																				0.00

**Notes.**

Diagonal elements—intracluster values, non-diagonal elements—intercluster values.

The percentage contribution of each trait towards total divergence was studied and presented in Table 7, where fruit yield per plant showed highest contribution towards genetic divergence (17.498%) followed by fruit length (11.390%), plant height (11.836%), days to 50% flowering (10.921%), fruit girth (9.297%), leaf length (8.672%), capsaicin content (8.554%) and leaf breadth (6.705%). While the traits number of main branch and days to maturity showed minimum contribution towards total divergence.

**Table 7:** Principal components values and percentage contribution of 11 different traits towards total divergence.

Traits	F1	F2	F3	F4	F5	Percentage contribution
Plant height (cm)	0.671	0.108	0.067	−0.166	−0.350	11.836
No of main branch	0.360	−0.069	0.027	0.124	0.883	3.415
Leaf length (cm)	0.574	0.620	0.173	0.358	−0.050	8.672
Leaf breadth (cm)	0.505	0.588	0.260	0.464	−0.078	6.705
Days to 50% flowering	0.645	−0.349	−0.313	0.347	−0.063	10.921
Capsaicin content (%)	0.570	−0.449	−0.223	−0.071	−0.148	8.554
Fruit length (cm)	0.658	0.310	−0.298	−0.456	0.048	11.390
Fruit girth (cm)	0.595	0.419	−0.373	−0.373	0.131	9.297
Fruit yield/plant (g)	0.816	−0.296	0.396	−0.207	0.034	17.498
No of fruits/plant	0.547	−0.513	0.634	−0.059	−0.009	7.864
Days to maturity	0.383	−0.395	−0.545	0.436	−0.058	3.846
Eigen value	3.805	1.849	1.334	1.096	0.961	–
Variability explained (%)	34.587	16.806	12.125	9.962	8.734	–
Cumulative (%)	34.587	51.393	63.518	73.480	82.214	–

**Figure 2:**
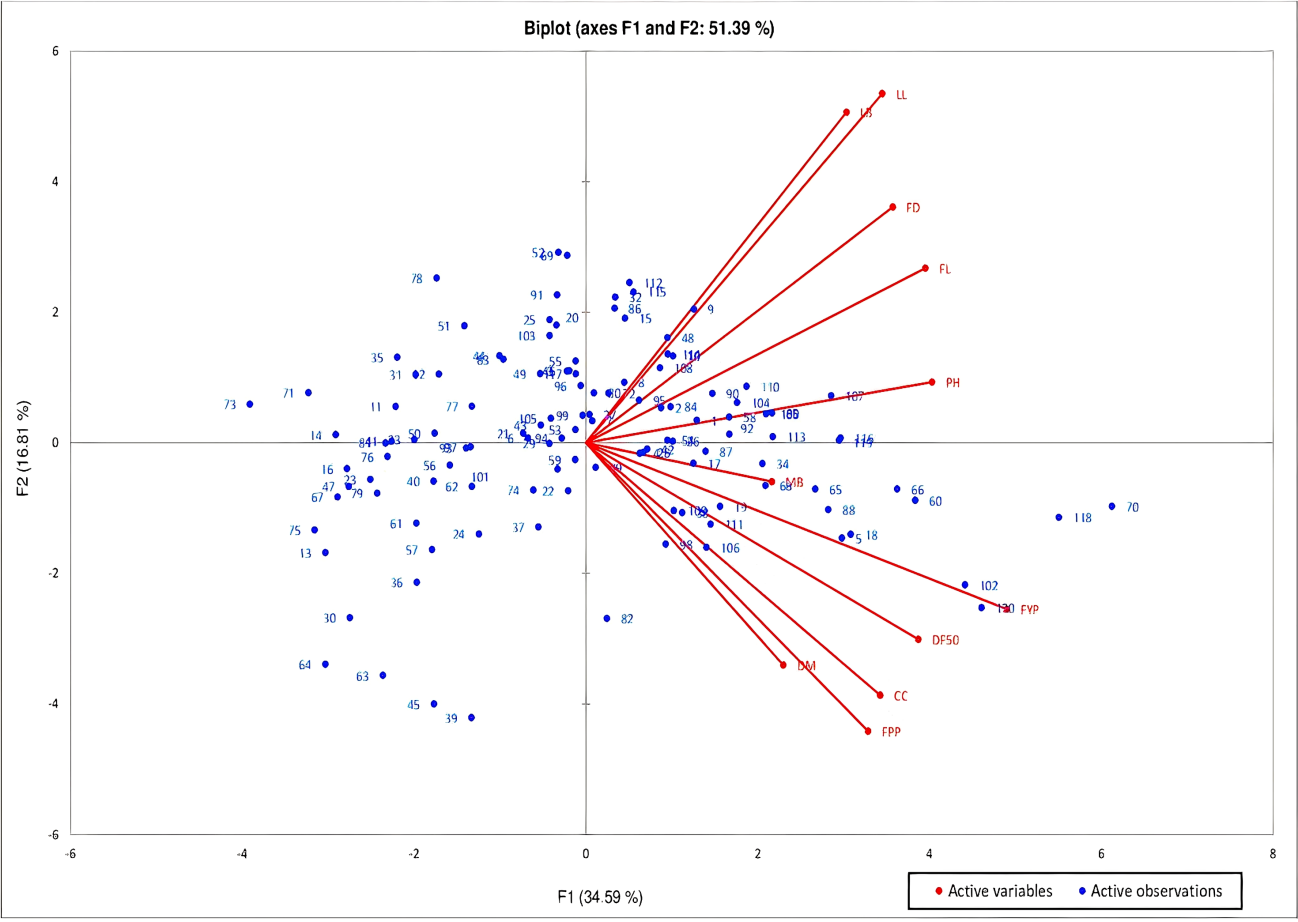
PCA biplot of *C. chinense* Jacq. genotypes based on PC1 and PC2 to determine the maximum contributor of variation at both axes.

Principal Components Analysis (PCA) was studied to determine the largest contributor of variation at different differentiation axis. PCA study revealed that about 73.48% of the variability is explained by the four principal components (F1, F2, F3 and F4) which have eigen value greater than 1. The first component added to 34.59% of the total variability, contributed by traits with high positive values *viz*-fruit yield per plant, followed by plant height, fruit length, days to 50% flowering, fruit girth, leaf length, capsaicin content, number of fruits per plant and leaf breadth (Fig. 2). The variability in this component is mostly associated with fruit characteristics. About 16.81% of the variability is added by the second component with strong positive contribution from traits-leaf length, leaf breadth, fruit girth and fruit length. The third and fourth components added to 12.13% and 9.96% of the total variability with strong contribution from the traits number of fruits per plant and fruit yield per plant for 3rd component and leaf breadth, and the traits days to maturity, leaf length and days to 50% flowering for 4th component, respectively. The biplot of the axes (PC1 and PC2) comprising about 51.39% of the variability showed that high PC1 and low PC2 is required for selection of genotypes with high fruit yield and capsaicin content. PC 1 showed positive correlation with all the studied traits while PC2 showed positive correlation with traits leaf length, leaf breadth, fruit length and girth and plant height.

## Discussion

Levene’s test (Levene, 1960) is a hypothetical test performed before ANOVA to test whether the studied environments have same or different variances. If the researched environments have distinct variants, then further research will be ineffective since this will result in large discrepancies across all of the settings. In the present study, Levene’s test of homogeneity of variance was reasonably met for ANOVA analysis to be performed. ANOVA study conducted for three years pooled data showed significant differences among the studied genotypes. It indicates that the genotypes have significant variation for the studied traits, which will be helpful in the selection of various traits for crop improvement.

The presence of genetic variability is the basis of all improvement programmes. High variation indicates the presence of higher variability for the traits which provides greater scope for improvement through pure line selection (Nahak et al., 2018). In the present study high variability parameters (*i.e.,* GCV, PCV) were obtained for fruit yield per plant, number of fruits per plant, capsaicin content, leaf breadth, fruit length and fruit girth. The present result is supported by the previous work of Ibrahim, Ganiger & Yenjerappa (2001), Manju & Sreelathakumary (2002), Nagaraju et al. (2018) and Negi & Sharma (2019). GCV and PCV estimates are done to determine the environmental effect on various traits. In the study it was observed that except for days to 50% flowering and days to maturity all the traits showed lower GCV estimates than PCV, indicating the great environmental influence on the expression of these traits and so selection should be made carefully considering the environmental changes, as suggested by Lal, Gupta & Dubey (2017). Singh & Kumar (2005) suggested that for more efficient selection process, heritability should be studied along with variability. According to Singh (2001) heritability of a trait is high when it is 80% or above, moderate when it ranged between 40–80%, and low when less than 40%. Based on these criteria seven traits showed high heritability, four traits showed moderate heritability, while low heritability was observed only for the trait number of main branch. Genetic advance is the enhancement in base population that can be possibly made from selection of a trait. Lal et al. (2013) suggested that high heritability along with high genetic advance is most preferred as these traits were controlled by additive gene action. Accordingly, five traits *viz*-fruit yield per plant, number of fruits per plant, capsaicin content, fruit length and fruit girth showed high heritability with high genetic advance and hence selection will be fruitful for these traits in early generations in ghost pepper. The present results were in agreement with the findings of Sharma, Semwal & Uniyal (2010), Meena et al. (2016) and Nagaraju et al. (2018) in *C. annum*. High heritability with high genetic advance was also observed for fruit length and girth in ghost pepper (Mena et al., 2019). Moderate heritability with low genetic advance observed for days to maturity (4.73%) indicated the occurrence of non-additive gene action, thereby making it difficult for improvement through direct selection. In such cases, improvement can be achieved using other breeding methods like-mutation, hybridization *etc*.

Correlation study for fruit yield per plant showed positive and highly significant correlation with nine traits, which is consistent with the reports of Mena et al. (2018), Devi et al. (2018) and Ozukum et al. (2019) in ghost pepper. The correlation study for capsaicin content was also consistent with the reports of Datta & Jana (2010) and Vaishnavi, Khanm & Bhoomika (2017) in different *Capsicum* species. Genotypic path study for fruit yield per plant has been studied because the results at phenotypic level may not provide appropriate results of direct and indirect relation of the component traits (Negi & Sharma, 2019). Direct positive effect of number of fruits on fruit yield of ghost pepper was also previously reported by Devi et al. (2018). Further, our results were similar to the findings of Sharma, Semwal & Uniyal (2010), Bijalwan & Mishra (2016) and Vaishnavi, Khanm & Bhoomika (2017) in different *Capsicum* species. Therefore, number of fruits per plant should be taken as important selection criterion for the ghost pepper improvement programme. Path study for capsaicin content showed direct effect from days to 50% flowering, which was similar to the reports of Mini & Vahab (2002) and Misra et al. (2010) in *C. annum*.

Mahalanobi’s D^2^ statistics (Mahalanobi, 1936), which is a most ideal tool for determining genetic divergence, was used in the present study where many clusters were formed irrespective of their place of collection. This formation of many individual clusters based on morphological characterization indicates the presence of sufficient genetic variability among them. In *C. annum*, Ajjappalavara (2009), Kumari et al. (2010) and Datta & Jana (2011) also observed formation of large number of clusters, indicating the presence of wide variability in the studied material. According to Patel et al. (2017) genotypes from highly divergent clusters should be used for the development of high-yielding varieties. Based on these criteria genotypes in clusters I and XIX would be more fruitful for producing segregates with great heterotic effects as they produced maximum distance between them. Further inter-cluster range observed in the present study was much higher than the results obtained by Yatung et al. (2014) in *C. annum*.

The study of different traits contribution towards genetic divergence is important with regards to selection and choice of parents for hybridization programme and is performed on the basis of D^2^ values. The trait like fruit yield per plant can be taken into consideration during selection of parents for hybridization as it showed highest contribution towards genetic divergence. It was followed by the traits fruit length, plant height, days to 50% flowering, fruit girth, leaf length, capsaicin content and leaf breadth. The maximum contribution by similar traits towards total divergence was also reported earlier by Hasan et al. (2015) in *C. annum*. PCA analysis was done to determine the largest contributor of variation at different differentiation axis (Sharma, 1988). According to Pandey & Bhatore (2018) higher the percentage contribution of traits towards divergence, the more effective it will be in recovering transgressive segregates using multiple cross over. Afuape, Okocha & Njoku (2011) suggested that for an effective breeding, only the components having eigen value greater than one should be taken for determining traits that can produce phenotypic difference. The PCA results in the present study showed majority of the variability was explained by the first four principal components, which was similar to the reports of Sarmah, Sarma & Gogoi (2018) for different chili varieties of Assam. In a recent study on *C. annum*, it was observed that the percentage contribution by the first two principal component was 28% and 19% respectively (Karim et al., 2022). In this respect, the current study showed high percentage contribution from the first two components, together accounting for 51.39%. Study by Azeez & Morakinyo (2011) showed that for selecting genotypes with high fruit yield and capsaicin content high PC1 components should be taken into consideration, which implies that the traits contributing more in the first principal component should be given preference. Accordingly, the traits-fruit yield per plant, plant height, fruit length, days to 50% flowering, fruit girth, leaf length, capsaicin content, number of fruits per plant showed positive contribution in the first principal component. Based on three-year detailed study, the genotypes-RRL-BJ-102, 120, 118, 70, 60, 66, 5, 18, 88, 65, and 106 are considered superior for capsaicin content and fruit yield and can be opted for large-scale evaluation.

## Conclusions

The two most significant characteristics of *Capsicum chinense* Jacq. crop in terms of economics are pungency and yield. Recent years witnessed a significant decline in popularity of this important crop due to the use of inferior planting material and lack of elite lines. It is therefore necessary to develop suitable elite lines with promising characteristics to meet the quality requirements. The current study aimed to identify the essential factors for selecting superior ghost pepper lines. Priority should be given to traits like high fruit yield per plant, fruit length, fruit girth, days to 50% flowering, days to maturity and plant height as these will either directly or indirectly influence varietal selection when developing a breeding programme for the development of ghost pepper. All these traits showed positive correlation, high heritability and genetic advance which are considered as important criteria for selection of elite lines. And based on percent contribution towards total divergence and PCA data, eleven genotypes (RRL-BJ-102, 120, 118, 70, 60, 66, 5, 18, 88, 65, and 106) were selected with high yield and capsaicin content. The lines can be opted for large-scale cultivation before going for variety recommendation and will be distributed for farmers’trial. To the best of our knowledge, this is the first detailed study on variability estimation and genetic divergence with three-year evaluation and incorporating large number of genotypes.

##  Supplemental Information

10.7717/peerj.15521/supp-1 Figure S1 C. chinense variants

10.7717/peerj.15521/supp-2 Data S1 Raw data for morphological and quality data of 120 germplasm of *Capsicum chinense* used in the study

10.7717/peerj.15521/supp-3 Table S1 Mean values of three years pooled data for 11 traits of *C. chinense*

## Abbreviations

ANOVA: Analysis of variance
GCV: Genotypic coefficient of variation
H: Heritability
Jacq.: Jacquin
PCA: Principal Components Analysis
PC1: Principal component 1
PC2: Principal component 2
PCV: phenotypic coefficient of variation
RCBD: Randomized Complete Block Design
SHU: Scoville Heat Unit

## Additional Information and Declarations

### Data Availability

The following information was supplied regarding data availability: The raw data are available in the Supplemental File.
