# Supplementary figures and images for: Estimation of genetic variation in yield, its contributing characters and capsaicin content of Capsicum chinense Jacq. (ghost pepper) germplasm from Northeast India
Source: PeerJ. 2023 Jun 22;11:e15521. doi: 10.7717/peerj.15521 (PMC10290828; doi:10.7717/peerj.15521)

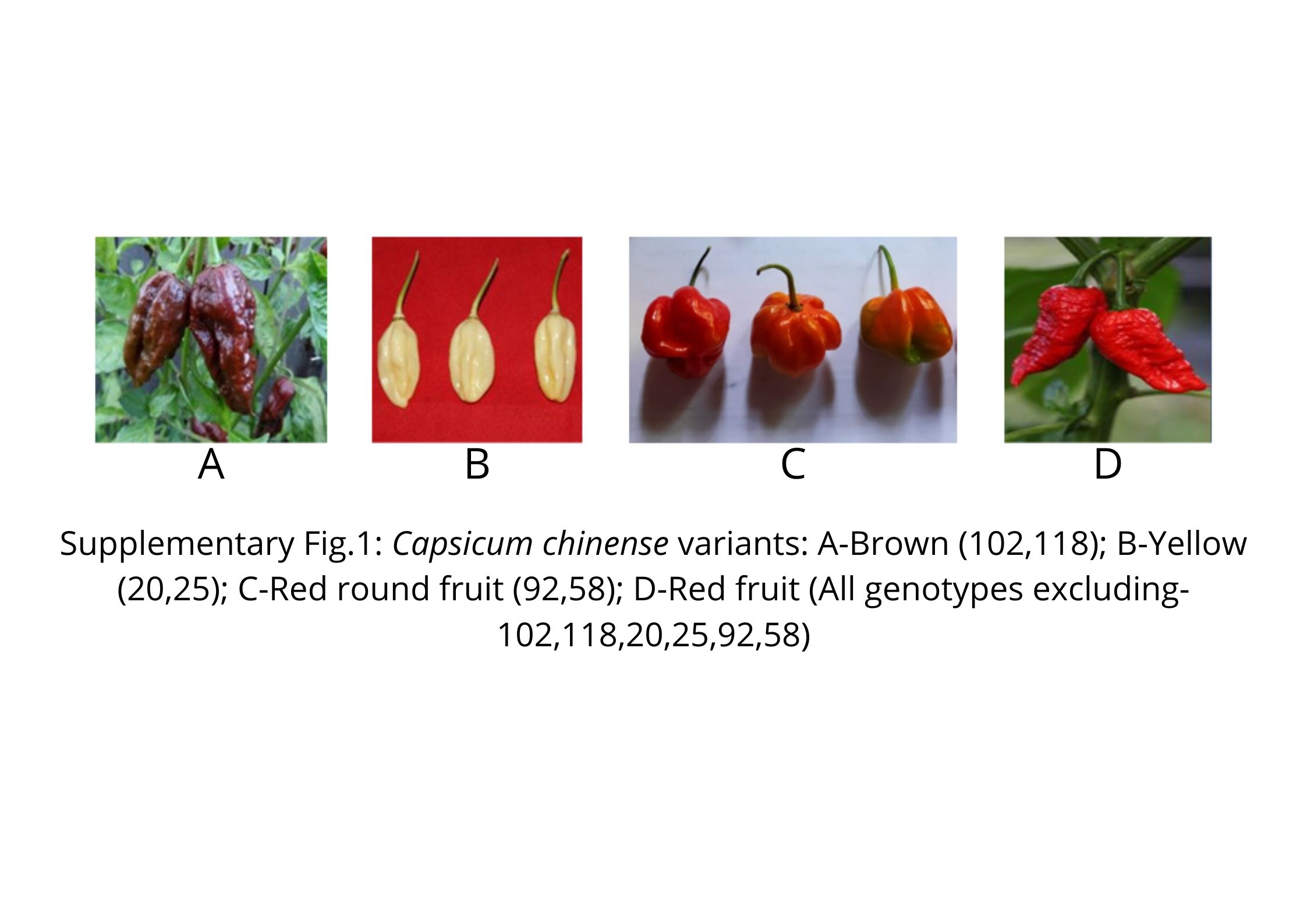

Supplement: Figure S1 [file peerj-11-15521-s001.jpg]
