# Supplementary material for: Estimation of genetic variation in yield, its contributing characters and capsaicin content of Capsicum chinense Jacq. (ghost pepper) germplasm from Northeast India
Source: PeerJ. 2023 Jun 22;11:e15521. doi: 10.7717/peerj.15521 (PMC10290828; doi:10.7717/peerj.15521)
Supplement: Table S1 [file peerj-11-15521-s003.docx]

Supplementary Table 1: Average climatic condition of experimental site during the study years

| Year | Temperature (°C) | | Humidity (%) | | Total rainfall (mm) | Wind speed (m/s) |
| --- | --- | --- | --- | --- | --- | --- |
|  | Minimum | Maximum | Minimum | Maximum |  |  |
| 2017 | 20.08 | 29.51 | 74.77 | 98.49 | 1781 | 0.84 |
| 2018 | 20.07 | 28.81 | 68.46 | 98.93 | 2099 | 0.82 |
| 2019 | 20.10 | 28.72 | 68.57 | 99.15 | 1972 | 0.90 |

Supplementary Table 2: Mean values of three years pooled data for 11 traits of *C. chinense* Jacq.

| Line No. | Plant height (cm) | No of main branch | Leaf length (cm) | Leaf breadth (cm) | Days to 50%flowering | Capsaicin content (%) | Fruit length (cm) | Fruit girth (cm) | Fruit yield/plant (g) | No of fruits/ plant | Days to maturity |
| --- | --- | --- | --- | --- | --- | --- | --- | --- | --- | --- | --- |
| 1 | 61.543 | 4.443 | 9.613 | 5.580 | 76.890 | 2.073 | 6.700 | 7.453 | 422.170 | 59.113 | 178 |
| 2 | 57.700 | 5.443 | 9.377 | 5.487 | 73.000 | 1.827 | 6.900 | 7.333 | 432.517 | 60.333 | 175.556 |
| 3 | 67.233 | 3.443 | 8.910 | 5.077 | 70.777 | 1.787 | 6.720 | 7.357 | 325.230 | 46.113 | 173.556 |
| 4 | 58.420 | 5.333 | 8.713 | 4.867 | 72.667 | 1.913 | 6.700 | 7.320 | 488.890 | 69.113 | 172.222 |
| 5 | 67.800 | 4.890 | 8.313 | 4.700 | 81.110 | 2.963 | 7.467 | 7.887 | 595.150 | 77.220 | 175.222 |
| 6 | 52.257 | 4.000 | 8.510 | 4.800 | 74.443 | 1.750 | 6.767 | 7.220 | 355.857 | 50.557 | 176.111 |
| 7 | 62.630 | 4.777 | 8.687 | 4.867 | 77.667 | 1.613 | 6.597 | 7.253 | 305.690 | 43.780 | 176.556 |
| 8 | 60.200 | 3.443 | 9.120 | 5.213 | 73.447 | 1.597 | 7.803 | 7.247 | 446.607 | 59.113 | 173.778 |
| 9 | 75.477 | 4.330 | 9.947 | 5.843 | 66.333 | 1.960 | 6.977 | 7.510 | 421.757 | 57.890 | 169.333 |
| 10 | 67.443 | 4.557 | 9.377 | 5.487 | 70.670 | 1.913 | 7.177 | 7.790 | 394.223 | 52.553 | 173.667 |
| 11 | 56.220 | 3.000 | 7.367 | 4.913 | 67.887 | 2.003 | 6.510 | 6.843 | 269.590 | 40.113 | 168.667 |
| 12 | 65.223 | 3.220 | 8.487 | 4.710 | 69.110 | 1.603 | 6.743 | 6.033 | 228.233 | 35.890 | 171.333 |
| 13 | 52.490 | 2.890 | 7.387 | 4.057 | 73.777 | 2.237 | 5.157 | 4.643 | 257.943 | 52.443 | 173.889 |
| 14 | 51.310 | 3.777 | 8.190 | 4.580 | 70.557 | 1.497 | 5.543 | 5.770 | 215.120 | 38.223 | 172.889 |
| 15 | 59.420 | 4.557 | 7.833 | 4.123 | 73.003 | 1.473 | 6.343 | 5.720 | 360.470 | 59.670 | 174.556 |
| 16 | 50.800 | 4.110 | 7.267 | 3.900 | 70.220 | 1.327 | 5.800 | 6.543 | 308.127 | 49.890 | 169.778 |
| 17 | 73.203 | 3.890 | 8.390 | 4.667 | 74.557 | 2.807 | 6.957 | 7.533 | 373.613 | 51.553 | 176.111 |
| 18 | 65.123 | 5.223 | 8.910 | 5.090 | 70.667 | 4.127 | 9.500 | 7.800 | 619.623 | 82.887 | 172.889 |
| 19 | 66.223 | 3.780 | 8.367 | 4.600 | 73.223 | 2.573 | 7.043 | 7.533 | 583.870 | 80.000 | 174.222 |
| 20 | 62.443 | 4.667 | 9.623 | 5.690 | 71.553 | 1.373 | 4.733 | 5.190 | 430.950 | 86.557 | 174.889 |
| 21 | 62.500 | 3.443 | 8.467 | 4.690 | 75.110 | 2.193 | 6.867 | 6.397 | 248.000 | 37.557 | 175.222 |
| 22 | 61.280 | 4.997 | 7.900 | 4.223 | 75.890 | 2.417 | 6.567 | 7.033 | 274.483 | 40.557 | 177.000 |
| 23 | 58.590 | 3.000 | 7.347 | 3.633 | 71.553 | 1.987 | 6.367 | 5.867 | 258.450 | 41.887 | 169.556 |
| 24 | 59.600 | 2.780 | 7.690 | 3.957 | 76.113 | 2.640 | 5.857 | 6.530 | 279.067 | 44.890 | 177.667 |
| 25 | 57.767 | 4.220 | 9.267 | 5.510 | 71.223 | 1.457 | 7.123 | 7.690 | 318.590 | 42.887 | 170.778 |
| 26 | 70.213 | 3.887 | 9.097 | 5.300 | 70.557 | 2.267 | 6.867 | 6.277 | 480.350 | 72.667 | 172.778 |
| 27 | 74.623 | 3.220 | 8.023 | 4.323 | 66.777 | 2.047 | 6.880 | 7.513 | 443.607 | 61.447 | 168.556 |
| 28 | 62.143 | 4.553 | 8.080 | 4.400 | 70.333 | 1.717 | 7.777 | 7.200 | 584.787 | 77.780 | 168.444 |
| 29 | 61.980 | 4.890 | 9.320 | 5.477 | 74.777 | 2.007 | 5.770 | 5.153 | 309.150 | 56.777 | 172.222 |
| 30 | 51.023 | 3.890 | 5.797 | 3.823 | 68.223 | 1.487 | 5.463 | 4.970 | 477.150 | 91.330 | 170.222 |
| 31 | 54.177 | 4.443 | 7.853 | 4.153 | 66.443 | 1.673 | 7.677 | 6.933 | 242.167 | 32.777 | 168.778 |
| 32 | 59.613 | 4.223 | 8.353 | 4.563 | 67.890 | 2.063 | 6.110 | 6.343 | 331.533 | 40.887 | 169.222 |
| 33 | 51.933 | 5.110 | 7.800 | 4.220 | 66.110 | 1.687 | 6.210 | 6.743 | 323.590 | 54.110 | 168.222 |
| 34 | 57.737 | 5.777 | 8.213 | 4.533 | 76.890 | 2.070 | 8.330 | 8.733 | 527.340 | 61.667 | 177.556 |
| 35 | 54.753 | 3.667 | 8.323 | 4.290 | 65.890 | 1.603 | 7.423 | 6.757 | 245.873 | 34.443 | 168.556 |
| 36 | 51.167 | 3.890 | 7.653 | 3.890 | 79.333 | 2.217 | 5.113 | 5.567 | 254.710 | 47.667 | 179.556 |
| 37 | 57.333 | 4.553 | 7.253 | 3.900 | 78.000 | 2.397 | 6.663 | 7.200 | 285.190 | 40.777 | 178.111 |
| 38 | 59.633 | 4.000 | 9.357 | 5.623 | 77.667 | 1.847 | 5.777 | 6.267 | 551.813 | 90.780 | 178.778 |
| 39 | 63.377 | 3.223 | 5.547 | 2.710 | 79.333 | 2.250 | 5.543 | 4.953 | 497.490 | 94.113 | 171.000 |
| 40 | 55.410 | 2.777 | 8.043 | 4.400 | 72.667 | 2.227 | 5.897 | 6.423 | 281.917 | 54.333 | 173.000 |
| 41 | 54.233 | 3.333 | 8.030 | 4.400 | 71.223 | 1.970 | 5.967 | 6.457 | 243.240 | 38.667 | 172.111 |
| 42 | 59.703 | 4.110 | 8.780 | 4.977 | 79.777 | 2.957 | 7.097 | 7.567 | 277.630 | 37.333 | 176.444 |
| 43 | 61.967 | 3.333 | 8.567 | 4.867 | 74.557 | 2.123 | 6.577 | 7.010 | 288.300 | 42.000 | 175.667 |
| 44 | 62.800 | 3.110 | 7.930 | 4.477 | 67.777 | 1.773 | 7.933 | 7.467 | 355.403 | 45.888 | 166.444 |
| 45 | 61.033 | 3.667 | 5.913 | 3.163 | 67.003 | 2.640 | 5.110 | 4.390 | 532.640 | 110.557 | 168.000 |
| 46 | 57.623 | 4.777 | 9.533 | 5.633 | 70.557 | 2.000 | 6.433 | 6.787 | 360.000 | 54.220 | 169.222 |
| 47 | 50.010 | 4.667 | 7.823 | 4.120 | 71.553 | 2.050 | 5.413 | 5.753 | 189.990 | 33.777 | 173.444 |
| 48 | 62.720 | 5.220 | 10.390 | 6.133 | 68.777 | 2.010 | 6.567 | 6.980 | 429.063 | 62.553 | 169.556 |
| 49 | 66.943 | 4.220 | 8.223 | 4.500 | 66.670 | 1.537 | 7.100 | 7.490 | 383.560 | 52.443 | 168.444 |
| 50 | 61.510 | 3.557 | 8.280 | 4.367 | 69.890 | 1.953 | 5.933 | 6.387 | 246.270 | 40.000 | 173.111 |
| 51 | 54.867 | 4.553 | 9.380 | 5.557 | 65.443 | 1.477 | 6.377 | 6.777 | 299.017 | 45.110 | 169.333 |
| 52 | 59.757 | 3.667 | 9.920 | 6.057 | 69.223 | 1.703 | 7.423 | 7.800 | 277.187 | 36.557 | 167.889 |
| 53 | 55.033 | 3.780 | 8.180 | 4.543 | 74.003 | 2.183 | 7.610 | 7.990 | 353.883 | 45.337 | 175.333 |
| 54 | 58.600 | 3.890 | 9.410 | 5.600 | 72.890 | 2.473 | 6.610 | 7.053 | 511.200 | 74.223 | 171.889 |
| 55 | 63.347 | 4.000 | 9.333 | 5.477 | 68.447 | 1.943 | 6.477 | 7.090 | 401.360 | 59.337 | 167.556 |
| 56 | 58.403 | 4.220 | 8.843 | 5.000 | 67.113 | 1.430 | 4.887 | 5.287 | 438.533 | 85.777 | 167.111 |
| 57 | 55.753 | 4.333 | 7.300 | 3.800 | 71.000 | 2.120 | 5.523 | 5.933 | 358.950 | 62.337 | 172.444 |
| 58 | 56.927 | 4.553 | 10.457 | 6.433 | 70.663 | 2.260 | 6.210 | 6.810 | 623.917 | 95.557 | 170.111 |
| 59 | 51.263 | 4.553 | 9.163 | 5.390 | 77.667 | 2.303 | 6.067 | 6.433 | 407.427 | 64.890 | 169.444 |
| 60 | 67.113 | 4.670 | 10.497 | 5.887 | 75.223 | 3.980 | 6.610 | 7.467 | 630.837 | 89.000 | 174.889 |
| 61 | 56.110 | 4.553 | 7.843 | 4.203 | 79.333 | 2.097 | 5.623 | 4.933 | 188.627 | 35.890 | 174.667 |
| 62 | 60.157 | 3.890 | 7.820 | 4.177 | 70.220 | 1.880 | 6.467 | 5.810 | 364.387 | 59.110 | 172.000 |
| 63 | 60.300 | 4.777 | 5.967 | 3.243 | 68.443 | 2.287 | 4.677 | 4.200 | 405.927 | 91.110 | 169.333 |
| 64 | 60.900 | 3.000 | 6.057 | 3.100 | 69.113 | 2.060 | 4.590 | 4.087 | 391.717 | 89.333 | 170.222 |
| 65 | 65.610 | 3.443 | 9.710 | 5.863 | 79.997 | 2.407 | 6.523 | 7.110 | 672.107 | 96.777 | 174.000 |
| 66 | 68.000 | 4.113 | 9.933 | 6.120 | 83.777 | 2.540 | 6.757 | 7.343 | 671.013 | 94.890 | 175.444 |
| 67 | 51.603 | 4.223 | 7.657 | 4.010 | 70.223 | 1.713 | 5.180 | 5.587 | 285.127 | 52.667 | 169.444 |
| 68 | 62.467 | 4.000 | 9.500 | 5.690 | 78.447 | 2.597 | 6.613 | 7.043 | 560.863 | 81.777 | 175.778 |
| 69 | 68.133 | 3.667 | 10.320 | 6.223 | 67.667 | 1.600 | 6.500 | 6.867 | 354.077 | 52.777 | 161.889 |
| 70 | 71.087 | 4.553 | 10.390 | 6.497 | 90.443 | 3.200 | 7.733 | 8.457 | 773.167 | 95.443 | 180.333 |
| 71 | 57.190 | 3.557 | 8.410 | 4.667 | 67.887 | 1.727 | 5.177 | 5.523 | 190.887 | 35.443 | 165.111 |
| 72 | 64.757 | 4.330 | 8.813 | 4.967 | 74.667 | 2.797 | 7.367 | 6.857 | 314.463 | 44.110 | 164.222 |
| 73 | 42.777 | 4.443 | 7.930 | 4.033 | 65.777 | 1.907 | 5.647 | 6.133 | 246.217 | 41.667 | 157.000 |
| 74 | 60.867 | 4.443 | 8.357 | 4.530 | 74.000 | 1.977 | 5.653 | 6.063 | 445.887 | 75.777 | 165.556 |
| 75 | 53.180 | 3.667 | 7.300 | 3.990 | 76.000 | 2.097 | 4.767 | 5.177 | 203.417 | 40.557 | 170.000 |
| 76 | 51.857 | 3.110 | 8.690 | 4.833 | 75.663 | 2.030 | 5.043 | 5.787 | 260.310 | 47.890 | 169.444 |
| 77 | 60.300 | 3.667 | 9.633 | 5.813 | 67.557 | 1.607 | 4.857 | 5.300 | 398.467 | 77.890 | 166.667 |
| 78 | 55.857 | 4.447 | 10.603 | 6.537 | 65.110 | 1.557 | 5.277 | 5.690 | 272.677 | 49.447 | 163.222 |
| 79 | 47.277 | 4.110 | 7.467 | 3.890 | 65.333 | 1.503 | 5.790 | 6.247 | 542.343 | 88.667 | 159.222 |
| 80 | 58.537 | 3.000 | 9.290 | 5.300 | 68.663 | 1.517 | 6.710 | 7.197 | 617.530 | 87.887 | 165.222 |
| 81 | 53.220 | 4.670 | 8.333 | 4.577 | 72.443 | 1.963 | 5.313 | 5.857 | 230.123 | 87.780 | 165.889 |
| 82 | 56.780 | 5.003 | 6.880 | 3.557 | 80.110 | 2.240 | 6.247 | 7.010 | 511.860 | 76.997 | 175.111 |
| 83 | 49.823 | 3.890 | 9.280 | 5.457 | 74.777 | 1.687 | 6.867 | 7.200 | 283.367 | 40.223 | 172.333 |
| 84 | 61.857 | 4.223 | 9.690 | 5.687 | 78.887 | 2.080 | 6.453 | 6.953 | 449.890 | 66.890 | 168.556 |
| 85 | 61.857 | 4.000 | 9.200 | 5.400 | 80.443 | 2.183 | 7.800 | 8.433 | 528.513 | 64.667 | 173.333 |
| 86 | 61.857 | 3.777 | 10.143 | 6.177 | 72.557 | 1.837 | 6.800 | 7.177 | 379.590 | 54.000 | 167.222 |
| 87 | 63.413 | 4.663 | 8.687 | 4.723 | 82.443 | 2.083 | 7.257 | 7.837 | 421.693 | 56.000 | 173.556 |
| 88 | 62.677 | 4.000 | 9.357 | 5.353 | 82.670 | 2.760 | 7.043 | 7.510 | 636.293 | 86.777 | 173.889 |
| 89 | 53.767 | 3.777 | 9.303 | 5.397 | 79.220 | 2.377 | 5.867 | 6.600 | 437.907 | 70.110 | 169.556 |
| 90 | 66.177 | 3.553 | 9.380 | 5.553 | 77.890 | 2.517 | 6.990 | 7.820 | 467.483 | 62.777 | 167.333 |
| 91 | 59.247 | 4.113 | 9.753 | 6.013 | 72.333 | 1.893 | 6.853 | 7.253 | 294.617 | 41.890 | 165.111 |
| 92 | 51.810 | 4.223 | 8.153 | 4.247 | 68.557 | 1.363 | 6.777 | 5.867 | 283.083 | 49.220 | 161.778 |
| 93 | 57.210 | 4.780 | 7.677 | 4.000 | 72.890 | 1.367 | 6.777 | 6.390 | 395.183 | 59.447 | 164.556 |
| 94 | 63.943 | 3.780 | 9.623 | 5.710 | 75.223 | 1.457 | 5.123 | 5.423 | 447.553 | 84.110 | 168.333 |
| 95 | 61.210 | 3.663 | 9.733 | 6.080 | 75.887 | 1.917 | 6.253 | 6.677 | 433.807 | 66.667 | 173.000 |
| 96 | 63.690 | 3.890 | 9.057 | 5.277 | 73.777 | 1.670 | 6.553 | 6.967 | 391.383 | 57.443 | 169.556 |
| 97 | 53.933 | 4.000 | 8.730 | 5.200 | 78.337 | 2.057 | 5.447 | 5.967 | 306.670 | 53.553 | 166.556 |
| 98 | 59.467 | 4.777 | 8.357 | 4.520 | 76.553 | 3.220 | 6.313 | 6.967 | 449.850 | 67.443 | 172.889 |
| 99 | 58.723 | 3.447 | 7.657 | 6.000 | 69.330 | 2.403 | 6.953 | 7.337 | 484.247 | 67.557 | 166.000 |
| 100 | 60.280 | 4.000 | 9.657 | 5.790 | 78.113 | 2.697 | 7.267 | 7.820 | 580.383 | 76.667 | 166.222 |
| 101 | 60.533 | 4.113 | 7.623 | 3.977 | 76.000 | 2.697 | 6.477 | 6.910 | 278.807 | 41.113 | 165.667 |
| 102 | 60.910 | 4.223 | 8.547 | 4.737 | 76.887 | 4.187 | 8.823 | 8.080 | 877.060 | 103.443 | 170.444 |
| 103 | 58.413 | 3.890 | 9.267 | 5.457 | 68.887 | 1.850 | 6.890 | 7.247 | 440.473 | 62.000 | 161.000 |
| 104 | 65.333 | 3.667 | 9.033 | 5.210 | 74.663 | 2.227 | 7.553 | 8.110 | 622.973 | 79.223 | 164.222 |
| 105 | 61.857 | 4.443 | 8.177 | 4.457 | 66.333 | 1.640 | 7.233 | 6.647 | 539.613 | 77.337 | 161.778 |
| 106 | 60.400 | 4.777 | 8.933 | 5.167 | 78.997 | 3.127 | 5.710 | 6.523 | 510.777 | 82.333 | 171.222 |
| 107 | 64.153 | 3.223 | 10.600 | 6.580 | 82.113 | 3.247 | 7.700 | 7.063 | 478.263 | 64.443 | 172.111 |
| 108 | 65.500 | 3.890 | 8.790 | 4.843 | 75.443 | 2.193 | 8.667 | 7.223 | 407.740 | 50.777 | 165.444 |
| 109 | 58.723 | 4.113 | 8.863 | 5.067 | 82.443 | 2.327 | 6.157 | 6.823 | 509.903 | 77.667 | 171.333 |
| 110 | 62.390 | 4.667 | 10.587 | 6.290 | 81.667 | 2.193 | 6.467 | 6.903 | 452.183 | 67.447 | 170.111 |
| 111 | 63.980 | 5.003 | 7.843 | 4.110 | 78.777 | 2.470 | 6.887 | 7.747 | 579.387 | 78.443 | 167.111 |
| 112 | 63.233 | 3.777 | 9.657 | 5.647 | 72.777 | 1.773 | 7.657 | 8.200 | 386.127 | 48.670 | 163.889 |
| 113 | 65.013 | 4.113 | 9.757 | 5.933 | 74.330 | 1.493 | 6.710 | 7.100 | 521.663 | 75.113 | 165.333 |
| 114 | 63.557 | 5.443 | 9.300 | 5.477 | 76.777 | 2.120 | 6.990 | 7.613 | 590.740 | 80.557 | 169.000 |
| 115 | 69.610 | 3.887 | 9.723 | 5.833 | 74.220 | 1.397 | 7.053 | 7.477 | 386.827 | 53.223 | 164.778 |
| 116 | 71.237 | 5.220 | 9.757 | 5.800 | 83.890 | 2.273 | 6.733 | 7.480 | 506.383 | 70.667 | 173.333 |
| 117 | 58.813 | 3.667 | 8.743 | 5.210 | 76.223 | 1.843 | 7.143 | 7.623 | 369.567 | 51.667 | 167.333 |
| 118 | 68.637 | 4.887 | 9.613 | 5.757 | 87.890 | 2.523 | 8.833 | 7.803 | 832.930 | 99.443 | 178.556 |
| 119 | 72.243 | 5.000 | 9.110 | 5.313 | 73.887 | 2.027 | 8.263 | 6.970 | 675.123 | 88.443 | 170.556 |
| 120 | 65.843 | 5.000 | 8.600 | 4.833 | 91.110 | 2.537 | 8.477 | 7.067 | 773.930 | 99.337 | 180.222 |
